# Cancer in Pregnancy: A 10-Year Experience in Shahid Sadoughi Hospital, Yazd, Iran

**Published:** 2013-09

**Authors:** M. Karimi-Zarchi, M. Ghane Ezabadi, S. Hekmatimoghaddam, M. Mortazavizade, S. Taghipour, M. Vahidfar, H. Vahedian, M. Forat, F. Shamsi, A. Miratashi-Yazdi

**Affiliations:** 1 Department of Gynecological Oncology, School of Medicine, Shahid Sadoughi University of Medical Sciences, Yazd, Iran;; 2 Shahid Sadoughi University of medical sciences, Yazd, Iran;; 3 Department of laboratory sciences, school of paramedicine, Shahid Sadoughi University of medical sciences, Yazd, Iran;; 4 Medical Oncologist, Azad University of Medical Science, Yazd Branch, Iran;; 5 Department of pathology, school of medicine, Shahid Sadoughi University of medical sciences, Yazd, Iran;; 6 Department of internal medicine, school of medicine, Shahid Sadoughi University of medical sciences, Yazd, Iran;; 7 Department of biostatistics and epidemiology, school of health, Shahid Sadoughi University of medical sciences, Yazd, Iran;; 8 Internal Medicine, Azada University of Medical Science, Yazd Branch, Iran

**Keywords:** cancer, pregnancy, outcome, Yazd, diagnosis, treatment, management

## Abstract

**Introduction:**

Although occurrence of cancer during pregnancy is rare, it leads to high morbidity and mortality in both mother and fetus. Recent trends in prolongation of child-bearing age have made cancer-associated pregnancies more frequent than past. As yet there are few documents concerning cancer and its related treatment outcomes during pregnancy. This study aimed at describing clinical characteristics of pregnant women with cancer in the Shahid Sadoughi hospital in Yazd, Iran.

**Materials and methods:**

Case series were reviewed retrospectively, which included 19 pregnant women diagnosed with cancer in Shahid Sadoughi hospital from 2002 to 2012. Data collected comprised demographics, pregnancy characteristics and outcomes, type of cancer, clinical stage, treatment and oncological.

**Results:**

From 17 pregnant women with cancer, 4 women had gynecologic cancers and 13 had non-gynecologic cancers. The Following tumors were observed: breast [6], acute myeloblastic leukemia [3], uterine cervix carcinoma [3], ovary [1], chronic myelogenous leukemia [1], lymphoma [1], papillary carcinoma of thyroid [1], and pseudopapillary carcinoma of pancreas [1]. The mean age of patients was 30.6 years, and the mean gestational age at diagnosis was 21.1 weeks. Surgical treatment was performed in 3 patients, 6 patients were treated by chemotherapy, and in 3 by both.

**Discussion:**

Although cancer during pregnancy is uncommon, it is considered an important problem due to unsuitable maternal and fetal outcomes and lack of standard management guidelines. Our cases represent examples of feasible or justifiable managements for them.

## INTRODUCTION

Cancer represents a major public health issue globally. In 2009 the American Cancer Society estimated that one in every four deaths in the United States was due to cancer, and that cancer was the second most common cause of death during reproductive years (1). The diagnosis of cancer during pregnancy is a rare condition with inherent poor prognosis (2). It poses challenges to the woman, her family and the medical team. As women tend to delay childbearing to third decade of life, the incidence of pregnancy-associated cancer is expected to increase (3). During the last decade, the age at first birth in Europe has increased by two years on average (4), reaching 30 years in some countries (5). This is why an association between cancer and pregnancy may be expected to occur more frequently. Management of cancer during pregnancy is a challenge, and its staging and therapeutic interventions must be performed carefully, bearing in mind the risk factors for both pregnant mother and the unborn child. The presenting symptoms of malignancy include lumps, nipple or vaginal discharge, fatigue, anemia, nausea, bone pain, rectal bleeding etc, which are often falsely attributed to gestational changes, resulting in delayed diagnosis (6).The most common cancers that occur in pregnancy include breast carcinomas, cervical cancers, Hodgkin’s disease, melanoma, leukemia and ovarian cancers (7). There are few numbers of documents concerning cancer and its related treatment outcomes and prognosis during pregnancy. The aim of this study was to describe the clinical characteristics of pregnant women with cancer in Shahid Sadoughi hospital in Yazd, Iran.

## MATERIALS AND METHODS

Case series in Shahid Sadoughi hospital were reviewed retrospectively, which included 17 pregnant women diagnosed with cancer from 2002 to 2012. Required data were collected from gynecology, surgery and oncology wards. Data from obstetrics and pediatric files were also added. We obtained demographics, pregnancy characteristics and outcomes, types of cancers, clinical stage, treatment and oncological outcome data for each case. Diagnosis of cancer was based on pathology reports which were assessed by a pathologist, and the staging was according to TNM system (Tumor size lymph, Node involvement, Metastasis). Quantitative data were assessed by Mann-Whitney U test using the SPSS 16. Kaplan-Meier curve was used to determine survival duration.

## RESULTS

Between 2002 and 2012, 17 pregnant women diagnosed with cancer were found. From all pregnant women, we had 4 gynecologic and 13 non-gynecologic malignancies. Mean age of the patients was 30.6 years. The mean gestational age at diagnosis for all patients, gynecologic and non-gynecologic malignancies were 21.1, 27 and 18.9 weeks, respectively. Cesarean section was the most common mode of delivery.

In our research breast cancer was the most common type of cancer during pregnancy. The Following tumors were observed: breast [6], acute myeloblastic leukemia (AML) [3], uterine cervix carcinoma [3], chronic myelogenous leukemia (CML) [1], ovary [1], lymphoma [1], papillary carcinoma of thyroid [1], and pseudopapillary carcinoma of pancreas [1]. Surgical treatment was performed in 23.5% of patients, 41.2% were treated by chemotherapy and 17.6% of them by both. From all patients with gynecologic malignancy, 3 cases had cervical and 1 case had ovarian cancer. Thirteen infants (76.7%) were born aliveand 4 fetuses (23.5%) were aborted. Early stage (defined as stages 1 and 2) was the most frequent (54.5%). Advanced stage (defined as stages 3 and 4) was found in 45.5 % of the patients. About 35.3% of mothers were cured completely, 35.3% are alive and being followed until now, and the remainder are died. Tables 1 and 2 show characteristics of the pregnant women with gynecologic and non-gynecologic malignancies, respectively. The mean survival duration was 12.3 ± 15 months in all patients, (8.2 ± 7.8 months in gynecologic and 25.5 ± 25.6 months in non-gynecological groups).

**Table 1:** Characteristics of patients with gynecological malignancies

Age (year)	Type of tumor	Stage	Gestational age (week)	Pregnancy No.	Fetus outcome	Treatment	Final situation

42	Squamous cell carcinoma of cervix	IIA	14	4	Abortion	Surgery	Alive
24	Adenocarcinoma of cervix	IIB	28	1	Cesarean section	Not permitted	Died
32	Papillary adenocarcinoma of cervix	IA2	34	2	Cesarean section	Surgery of the end of pregnancy	Alive
16	Adenocarcinoma of ovary	IIIA	32	1	Cesarean section	Surgery and chemotherapy	Alive

**Table 2:** Characteristics of patients with non-gynecological malignancies

Age (year)	Type of tumor	Stage	Gestational age (weeks)	Pregnancy No.	Fetus outcome	Treatment	Final situation

37	Pseudopapillary carcinoma of pancreas	II	12	1	Cesarean section	Surgery	Alive
32	Breast cancer	IIIB	15	2	Normal vaginal delivery	No treatment during pregnancy	Alive
39	Breast cancer	IIA	12	3	Abortion	Surgery	Alive
33	Breast cancer	IV	20	1	Cesarean section	Surgery and chemotherapy	Alive
31	Breast cancer	IIA	32	3	Cesarean section	Surgery and chemotherapy	Alive
36	Breast cancer	IIA	20	6	Normal vaginal delivery	chemotherapy	Alive
28	Breast cancer	C	20	6	Cesarean section	chemotherapy	Alive
34	Lymphoma	III	33	6	Normal vaginal delivery	No treatment during pregnancy	Alive
29	Papillary carcinoma of thyroid stage I		20	2	Cesarean section	Nothing	Alive
44	Chronic myelogenous leukemia	-	28	7	Normal vaginal delivery	chemotherapy	Alive
26	Acute myeloblastic leukemia	-	8	2	Abortion	chemotherapy	Died
26	Acute myeloblastic leukemia	-	32	1	Cesarean section	chemotherapy	Died
22	Acute myeloblastic leukemia	-	4	1	Normal vaginal delivery	chemotherapy	Died

**Figure 1:**
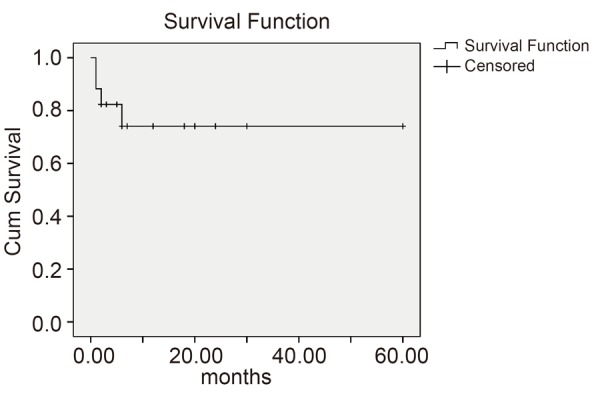
Survival in months for all patients.

Figure 1 shows the Kaplan-Meier survival estimates for all of the patients.

## DISCUSSION

Cancer is a major public health problem in world and Iran (8). From 2003 to 2010, 413,591 cases of cancer were recorded in Iranian cancer registry center. From all, 182,019 persons were women (9). Many (45.5%) of our cases were diagnosed in advanced stages, probably due to the unsuitable screening or to high estrogen levels from pregnancy which can aggravate cancers by suppression of the immune system during pregnancy (10). The most common cancers during pregnancy in other studies were breast and cervical cancers, Hodgkin’s disease, melanoma, leukemia and ovarian cancers (7). In our study the most common cancers were breast cancer, cervical cancer and AML. Differences in incidence and screening program may be the explanation for rarity of other types of cancers in our society in comparison with others. The presenting symptoms of malignancy were lumps, nipple or vaginal discharge, fatigue, anemia, nausea, bone pain or rectal bleeding, all being often falsely attributed to gestation, resulting in delayed diagnosis (6). In addition, exposure to ionizing radiation is restricted to prevent risk of damage to fetus. So, diagnosis of cancer during pregnancy is very difficult. In our study most of the treatments were in the second and third trimesters. As organogenesis is complete at the end of the first trimester, toxic effects leading to overt teratogenesis during the second and third trimesters are rare (11).

In our study the most common gynecological cancer was cervical cancer. In most countries, carcinoma of the cervix is the most common gynecological cancer in pregnancy, too (6). The reported incidence of cervical cancer in pregnancy is 1.2 per 10,000 pregnancies (12). The introduction of cervical screening programs has resulted in a reduction in incidence of cervical cancer, as well as in earlier detection of the disease. Unfortunately, advanced cervical cancer is still one of the most common cancers in developing countries (13). Cervical cancer is staged clinically, which relies on clinical examination. In pregnant women with cervical cancer, clinical examination can be more difficult. According to the recent studies, if the cancer is diagnosed in the first trimester, the woman is advised to terminate the pregnancy. If it is diagnosed in the second trimester, the treatment is delayed until the fetus is matured (6). In our research, cases of cervical cancer were diagnosed in the second and third trimesters. One of them with stage IIB and involvement of parametrium did not permit to do any supportive treatment during pregnancy. The mode of delivery was cesarean section and newborn baby was healthy. After the delivery radiotherapy and chemotherapy was used for mother, but because of the metastasis of tumor, the mother died after 6 months. Now the baby is seven years old and healthy. For the other patient with stage IIA and without involvement of parametrium, radical hysterectomy was the choice. Fetus was aborted and after that, chemotherapy and radiotherapy was started. Now after 6 years the mother is treated and under follow-up. In the other case with stage IA2 papillary adenocarcinoma, ending to the pregnancy with cesarean section in week 34 was undertaken. After the delivery radical trachelectomy was performed for mother, and after recurrence of tumor, radical hysterectomy, chemotherapy and radiotherapy were done (14).

The other gynecological cancer in our cases had been adenocarcinoma of ovary. Ovarian cancer in pregnancy is rare; it is said to occur in 1:15,000 to 1: 32,000 pregnancies (15). The diagnosis of ovarian cancer in pregnancy is not always easy (6). Ovarian tumors that have been diagnosed in premenopausal period are mainly in early stage and lower grade and could be treated by conservative surgery (16). Pregnancy may result in complications in ovarian tumors such as torsion, hemorrhage or rupture (12). Pregnancy by itself doesn’t seem to worsen the prognosis of the cancer. The timing of surgery for ovarian masses in pregnancy is usually delayed to the early second trimester, as intervention in the first trimester often results in miscarriage (6). Pregnant women in advanced stage of ovarian cancer seem to have poor prognosis. Chemotherapy is not contraindicated during the second or tired trimester, but the choice of couple must be considered (17). The case in our study was treated by surgery and chemotherapy. The mode of delivery was cesarean section. After delivery, surgery and chemotherapy were continued for mother. Now the mother and child are healthy. After 18 months, she is pregnant again (18).

Approximately 3% of breast cancers are diagnosed during pregnancy (19). The median reported maternal age at the time of diagnosis is 33-34 years, and the median gestational age is at 17-25 weeks (7). The histopathology features are similar to those who are not pregnant (20). The prospective cohort from the University of Texas (MDACC) reported that 84% of the tumors are poorly differentiated (21). Due to the physiological changes of pregnancy, breast cancer diagnosis may be delayed from 2 to 18 months compared to non-pregnant women (15). Approximately 65-90% of pregnant patients are diagnosed at stage II and III compared to 45-65% of non-pregnant patients (21). Modified radical or conservative surgery with axillary lymph node biopsy (SLNB) is still controversial (22). Chemotherapy during the first trimester is contraindicated and should be postponed (6). Retrospective studies have shown that survival of women with breast cancer during pregnancy is worse, regardless of the age of the mother (23). Otherwise, the prognosis is similar to that of non-pregnant patients of similar stage, grade and hormonal status (20). In our study we had 6 cases of breast cancer. Two of them were treated by surgery and chemotherapy and are continuing their treatments until now. One of the cases was operated in the 12th week and her fetus was aborted, but after 2 years, she is pregnant again. She was advised to abort. The other case of our survey was diagnosed after delivery, and now after the surgery she is continuing her treatment by radiotherapy and chemotherapy. We guess that tumor was created during pregnancy or before, but due to lack of exact diagnosis, she was not treated during pregnancy. The stage of tumor in this case was IIIB, and her fetus was born healthy. One of the other cases is at the gestational age 20 weeks, has received chemotherapy, but delivery has not happened as yet.

Acute myeloblastic leukemia (AML) represents two-thirds of all acute leukemias which occur during pregnancy (24). Although the diagnosis may be delayed due to vague symptoms that can be attributed to pregnancy (i.e. fatigue, anemia), immediate initiation of the therapy is required upon diagnosis. When the diagnosis is made during the 1st trimester and treatment is initiated, there are high rates (nearly 50%) of adverse fetal outcomes (25). During the 2nd and the 3rd trimester the same induction and consolidation regimens as for non-pregnant patients are used (6). Leukemia and lymphoma are the second most frequent malignancies, after melanoma that metastasizes to the placenta of the fetus (26). In our study we had three cases of AML. All of the patients with AML were treated by chemotherapy and finally died. We also had a CML case that is now pregnant at week 28.

Non-Hodgkin’s lymphoma during pregnancy is rare and few reports exist in the literature. When the diagnosis is made during the 1st trimester, chemotherapy should be initiated (6). Prognosis for pregnant patients with non-Hodgkin's lymphoma does not seem to be inferior to that of non-pregnant patients (27). We had one case of diffuse large B-cell lymphoma that was diagnosed 1 month after delivery. After the delivery the choice of treatment for her was chemotherapy, and she is under treatment until now. We guess that tumor was created in pregnancy or before, but due to loss of diagnosis a treatment was not used for this patient during pregnancy. Thyroid cancer has been reported to occur with an incidence of 14 cases per 100,000 pregnancies (28). For all patients with malignant nodules or with nodules that grow rapidly, surgery should be offered during the 2nd trimester of pregnancy (6). Patients with follicular or early stage papillary thyroid cancers are candidates for delayed surgical intervention, as these lesions usually do not progress rapidly (6). There have not been reported higher rates of locoregional relapse, or decreased survival in pregnant thyroid cancer patients when compared with non-pregnant patients (29). We had one case of papillary thyroid carcinoma that had received iodine therapy and surgery before the diagnosis of pregnancy. In the eight week of pregnancy it was diagnosed and iodine therapy was stopped. Now her child age is 20 weeks, and we are following her. In our study we had one case of mucinous cystadenoma of pancreas. Surgery was used for this patient at the week 12. The mother and her infant are healthy now.

Pregnancy affects the clinical presentation, evaluation, treatment, and prognosis of patients with gastrointestinal cancer. Pregnant patients may present with advanced gastrointestinal cancer as a result of delayed diagnosis, in part because of difficulty in differentiating signs and symptoms of cancer from signs and symptoms of normal pregnancy (30). The approach to cancer surgery and chemotherapy must be modified in pregnant patients to minimize fetal and maternal risks. Because of these factors, women who develop gastrointestinal cancer during pregnancy seem to have a poor prognosis (30).

## CONCLUSION

Although cancer during pregnancy is uncommon, it is a grave problem due to unsuitable maternal and fetal outcomes and lack of standard management guidelines. Since delay in diagnosis of cancer causes worse prognosis, it is very important to diagnose it in early stages. Termination of pregnancy, delay in maternal treatment and iatrogenic preterm delivery are frequently applied strategies in the management of pregnant cancer patients. Current knowledge relies mainly on experiences from small case series on cancer in pregnancy and several case reports. So, more studies and longer follow up may be useful in development of standard guidelines for its management.
